# Impact assessment of e-trainings in occupational safety and health: a literature review

**DOI:** 10.1186/s12889-023-16114-8

**Published:** 2023-06-20

**Authors:** Mohammad Mahdi Barati Jozan, Babak Daneshvar Ghorbani, Md Saifuddin Khalid, Aynaz Lotfata, Hamed Tabesh

**Affiliations:** 1 Department of Medical Informatics, School of Medicine, Mashhad University of Medical Sciences, Mashhad, Iran; 2 Department of Foreign Languages, Iran University of Science and Technology, Tehran, Iran; 3 Department of Applied Mathematics and Computer Science, Technical University of Denmark, Kongens Lyngby, Denmark; 4 School Of Veterinary Medicine, Department Of Veterinary Pathology, University of California, Davis, USA

**Keywords:** Occupational injuries and accidents, Preventive occupational health interventions, Online occupational safety and health training

## Abstract

**Background:**

Implementing workplace preventive interventions reduces occupational accidents and injuries, as well as the negative consequences of those accidents and injuries. Online occupational safety and health training is one of the most effective preventive interventions. This study aims to present current knowledge on e-training interventions, make recommendations on the flexibility, accessibility, and cost-effectiveness of online training, and identify research gaps and obstacles.

**Method:**

All studies that addressed occupational safety and health e-training interventions designed to address worker injuries, accidents, and diseases were chosen from PubMed and Scopus until 2021. Two independent reviewers conducted the screening process for titles, abstracts, and full texts, and disagreements on the inclusion or exclusion of an article were resolved by consensus and, if necessary, by a third reviewer. The included articles were analyzed and synthesized using the constant comparative analysis method.

**Result:**

The search identified 7,497 articles and 7,325 unique records. Following the title, abstract, and full-text screening, 25 studies met the review criteria. Of the 25 studies, 23 were conducted in developed and two in developing countries. The interventions were carried out on either the mobile platform, the website platform, or both. The study designs and the number of outcomes of the interventions varied significantly (multi-outcomes vs. single-outcome). Obesity, hypertension, neck/shoulder pain, office ergonomics issues, sedentary behaviors, heart disease, physical inactivity, dairy farm injuries, nutrition, respiratory problems, and diabetes were all addressed in the articles.

**Conclusion:**

According to the findings of this literature study, e-trainings can significantly improve occupational safety and health. E-training is adaptable, affordable, and can increase workers’ knowledge and abilities, resulting in fewer workplace injuries and accidents. Furthermore, e-training platforms can assist businesses in tracking employee development and ensuring that training needs are completed. Overall, this analysis reveals that e-training has enormous promise in the field of occupational safety and health for both businesses and employees.

## Introduction

Occupational injuries and diseases are among the most serious public health issues [1]. According to the most recent International Labor Organization report (2017), over 2.78 million workers die each year as a result of occupational accidents and work-related diseases [2]. The most serious negative consequences of occupational accidents and injuries are long-term disabilities [3] reduced ability to perform job duties [3–5], early retirement [3], medical care expenditure [4, 5], absenteeism [4–6], presenteeism [4, 5], and death [3]. These cost the global economy 3.94% of the global Gross Domestic Product (GDP) [2]. In various countries, these costs range from 1.8 to 6% of GDP [3].Treatment and preventive interventions are two types of interventions used to reduce occupational diseases and injuries, as well as the negative consequences of these events [7].

Preventive interventions in occupational health aim to change the work condition to prevent occupational accidents and reduce their harmful effects. There are three types of preventive interventions: primary preventive interventions, secondary preventive interventions, and tertiary preventive interventions [7]. Primary preventive interventions aim to create conditions that will help to prevent occupational disease and injury. In other words, these interventions aim to eliminate or reduce workers’ exposure to workplace hazards. Secondary and tertiary preventive interventions attempt to prevent disease or injury progression in the post-accident steps [7]. The primary preventive interventions are divided into three types: Environmental interventions, clinical interventions, and behavioral interventions are the first three [8]. Environmental interventions attempt to eliminate the causes of occupational accidents by altering work methods, equipment, and physical space [9]. Clinical interventions (for example, pre-employment medical examinations [10]) use therapeutic methods to prevent disease [8]. Behavioral interventions aim to change workers’ behavior in order for them to be safer at work [8].

Many developing and developed countries implement occupational safety and health programs (OSH) for workers due to the importance of safe behavior in reducing the costs of occupational accidents and their negative consequences [11]. The most important component of the OSH program has been introduced as education [12–16]. The World Health Organization (WHO) has also identified worker, employee, and occupational medicine specialist training as a key component in improving worker health [17].

The two main approaches in occupational education are class-based education and e-learning. Simple and low-cost solution [18–24], greater convenience [21], availability [18, 21, 22, 24], high acceptance among the workforce [25], enhanced self-management and adherence in the target population [26–28], primary source for health-related information [29, 30], internet availability for users [31–33], mobile phone availability for users [34, 35], ability to use a personalized approach [23], flexibility to fit the users’ schedules [33], reach large numbers of participants [23, 33] and prefer technology-enhanced educational programs [29] are the most important reasons that have made e-learning as a suitable alternative to traditional and class-based education.

In the last decade, studies [20, 36–39] have been conducted to evaluate the provision of online and personalized occupational health and safety training content. Systematic reviews have been conducted on the impact of occupational health and safety e-training in limited cases [23, 40–42], such as limiting studies to a geographical area [41], an occupational safety and health problem [23, 40, 42], a type of intervention [23], and etc. The limitations of systematic reviews did not have the comprehensive outlook on e-training role in the behavioral change and improve health. Given these relevant premises, the goal of this study is to conduct a systematic review of published studies that have used e-training to reduce occupational accidents through the end of 2021.

With the increasing use of technology in the delivery of training programs, e-learning has become a popular mode of training delivery with several potential benefits, such as flexibility, accessibility, and cost-effectiveness. This study seeks to evaluate the impact of e-trainings in occupational safety and health by conducting a comprehensive literature review. The findings of this study contribute to the ongoing discussions on how technology can be leveraged to improve workplace safety measures and reduce accidents and injuries. Moreover, it is essential to understand the effectiveness of e-training programs compared to traditional training methods and identify best practices for developing effective e-training programs. This study aims to fill this gap in current knowledge by providing a comprehensive analysis of the existing literature on e-trainings in occupational safety and health.

This study has two primary objectives: providing up-to-date details on online occupational health and safety training interventions and offering recommendations, discussing research gaps and challenges in online occupational health and safety training interventions.

## Materials and methods

As a paper selection methodology, the Preferred Reporting Items for Systematic Reviews and Meta-Analyses (PRISMA) [43] was utilized, which involves four phases: identification, screening, eligibility, and included. The first phase sets the search technique and databases used in the search. The title, abstract, and full text of the publications are assessed in the following steps, based on the inclusion-exclusion criteria set for the systematic review. The included articles are then determined. The qualitative synthesis is completed after the finalization of the included articles.

### Identification phase: search strategy

A computer-based literature search in the PubMed and Scopus databases was carried out. These databases were searched until the end of 2021. The search used a combination of text terms and a hierarchically regulated vocabulary that was tailored to each database. The text terms were divided into workplace safety and health (Group 1) and e-training (Group 2). These groups were joined together with “AND.“ Group 1 terms included occupational health, occupational safety, workplace health, and workplace safety. e-Training, e-Education, online training, online learning, mobile training, and mobile education were all included in Group 2. The terms from each of the three categories were then joined together with “OR.“

### Screening and eligibility phase

Determining the inclusion and exclusion criteria and the screening process are two important activities of this phase.

#### Inclusion/exclusion criteria

The inclusion criteria for this study involved educational interventions aimed at improving occupational safety and health among workers (Intervention) and published in English (Language) by the end of 2021 (Publication period). The interventions were designed for workers (Population) and delivered educational content through web or mobile-based platforms (Technology). The final inclusion criterion was that the interventions evaluate primarily outcomes related to occupational safety and health.

However, certain types of studies were excluded from the analysis. The interventions that were considered included those which examined multiple components or did not isolate the impact of training as a specific intervention component. The study population was also limited to excluding students, health professionals, disabled workers, military personnel, and drivers (Population). Outcome measures related to mental health, sleep, stress, and addiction were also excluded (Outcome). Similarly, studies utilizing 3D animation, Virtual Reality, Virtual game-based simulation, and 360-degree panoramas were excluded (Technology). Lastly, studies forced to use online education due to Coronavirus Disease 2019 (COVID-19) conditions were not considered.

#### Screening process

Two reviewers independently reviewed titles, abstracts, and full texts based on inclusion/exclusion criteria. Disagreements between the two reviewers on the inclusion/exclusion of an article were settled by consensus and, if required, by a third reviewer.

### Included phase: analysis

A meta-analysis of the impact of e-training on enhancing occupational safety and health is impossible due to the different nature of the literature on the type of treatments, study design, primary and secondary outcomes, and evaluation approaches. As a result, this study explains the nature of the included studies’ implementation practice models in order to highlight the studies’ strengths and limitations and make recommendations for future research.

The comparative analysis method is used for the analysis and synthesis in order to extract the themes emerging from the evidence [44]. This method, like other qualitative data analysis methods, involved coding data into themes and then categorizing and drawing conclusions based on them. These codes contained a concept associated with that part of the article from which the code is extracted. To maintain consistency and create non-overlapping code sets, a clear definition was provided for each code [45]. The qualitative content analysis process includes eight steps: data preparation, reading the article carefully several times to obtain a sense of the whole, determining the critical information of each part of the article (transcripts), defining the unit of analysis using themes, development of coding scheme to organize data, coding the entire article based on the developed coding scheme, conclusion based on the coded data, and describing and interpreting the findings [45]. Two reviewers independently read the articles in-depth and coded them. The reviewers and the lead author compiled the obtained results, and if there were any differences, they tried to resolve them. The disagreements were resolved by discussion.

Each article was reviewed to extract methods, parameters, and the purpose of evaluating e-training. The following codes were extracted for each article: the purpose of study, study design, population study, the unit of allocation (workplace/individual), country, primary/secondary outcomes, platform, educational content structure, and evaluation of the study.

## Result

**Fig. 1:**
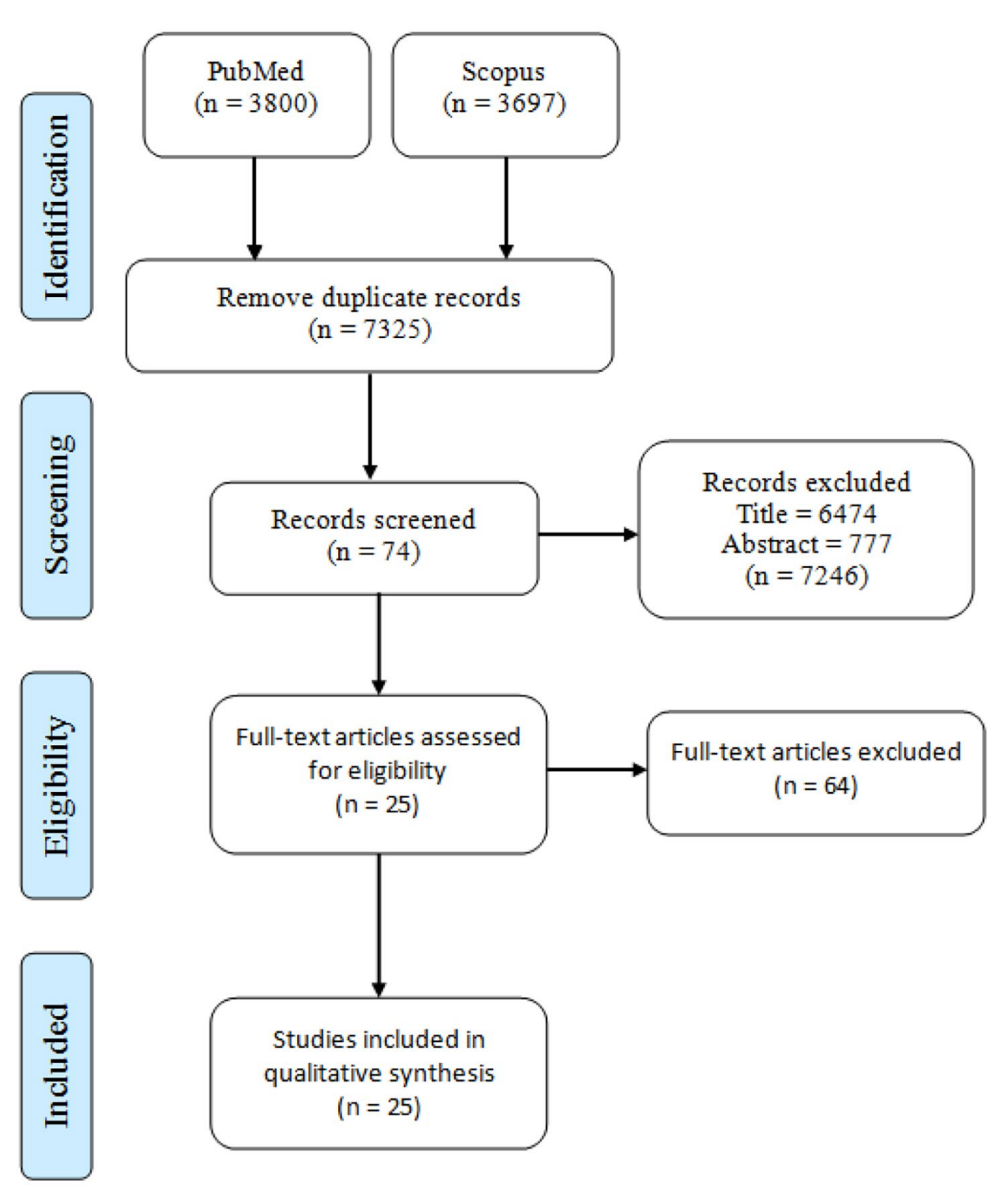
PRISMA flow diagram

The computer-based literature search yielded 7,497 articles, of which 7,325 were identified after removing 172 duplicate papers. Six thousand four hundred articles were removed during the title review phase and 777 articles were removed during the abstract review phase. 64 articles were removed during the title review phase. Finally, 25 articles met the criteria for inclusion. Figure 1 depicts PRISMA flow diagram of this systematic review study. The articles included in this review are listed in Table 1 in appendix.

**Fig. 2:**
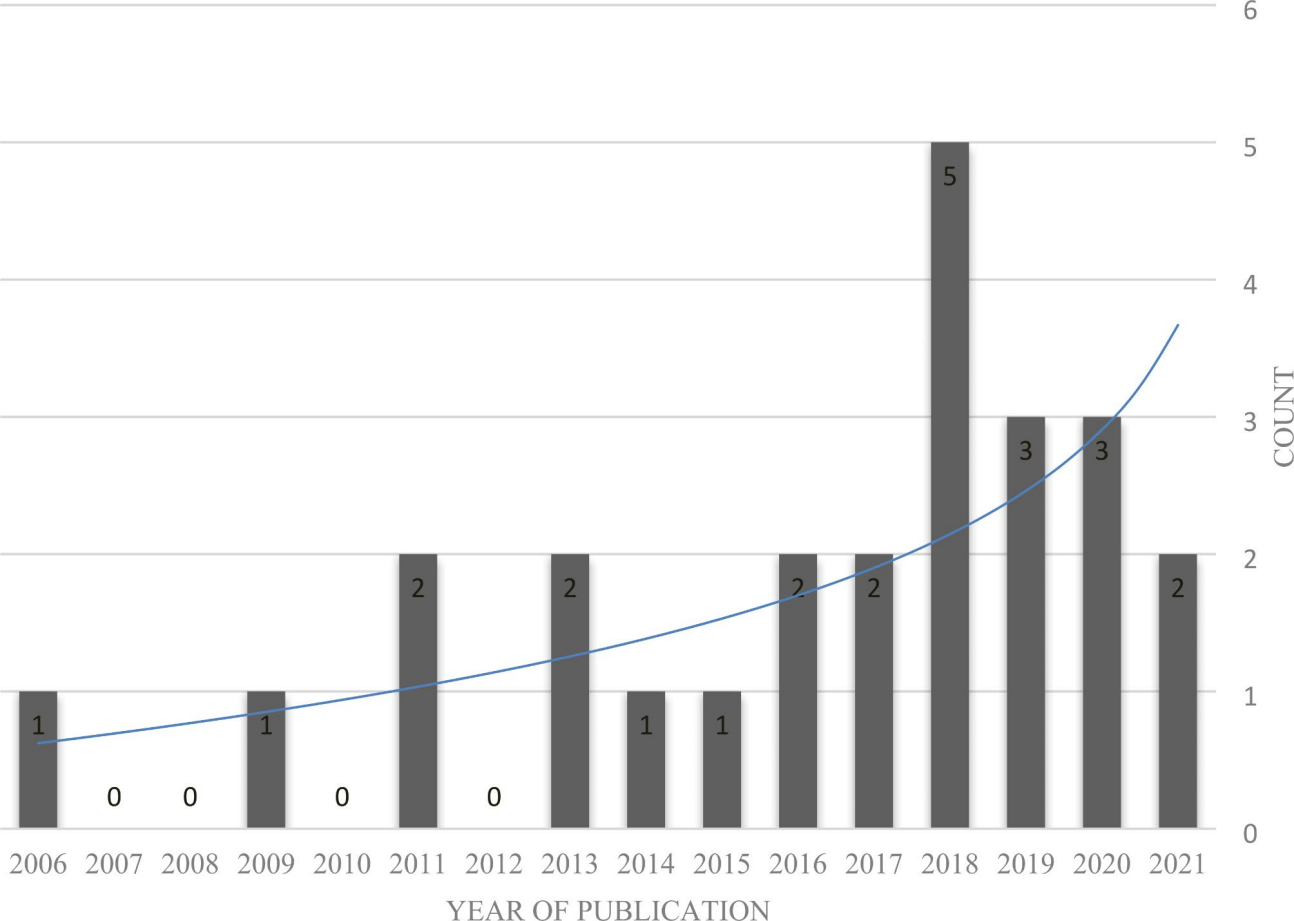
The number of the included studies based on the publication year

**Fig. 3:**
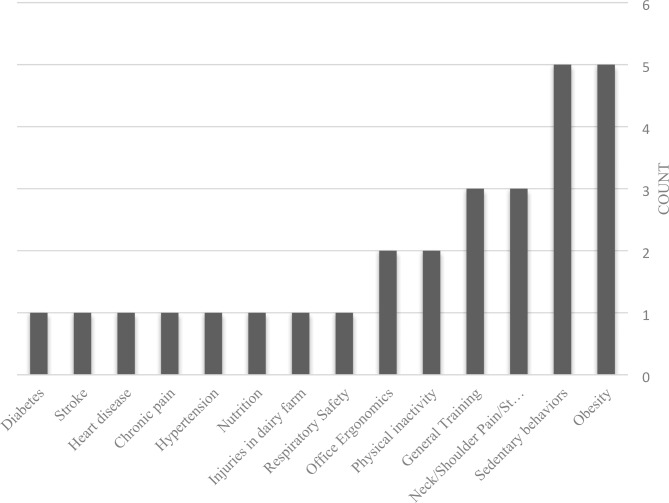
The number of the included studies based on the topic

**Fig. 4:**
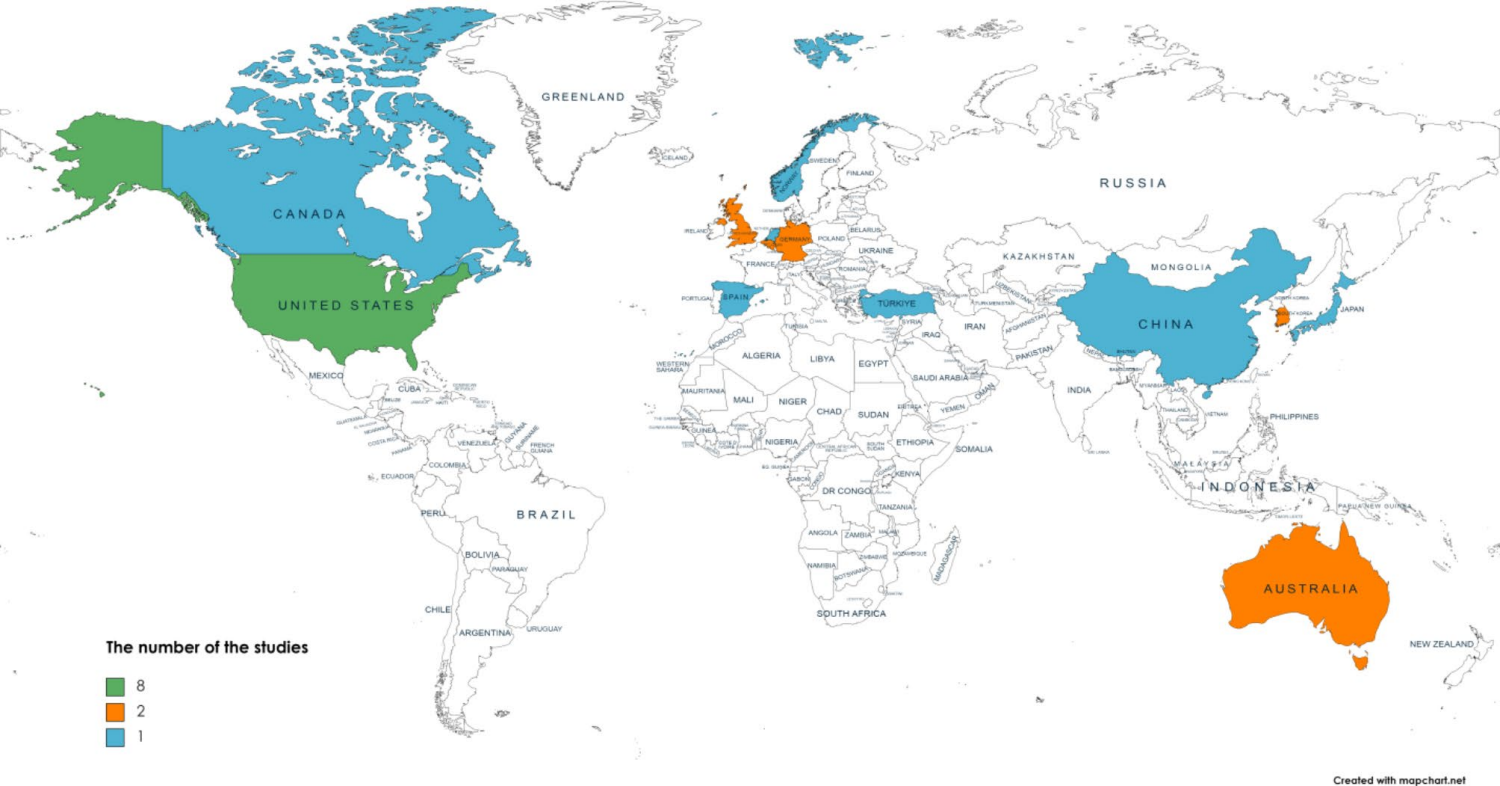
The number of the included studies based on each country. USA (n = 8); Australia, South Korea, UK, Germany, Belgium (n = 2), Japan, Turkey, Norway, Canada, Spain, China, Netherlands (n = 1)

The summary of the evaluation of the articles is shown in Table 1. E-training has been used in 9 areas of occupational safety and health: sedentary behaviors, obesity, neck/shoulder pain/stiffness and Low Back Pain (LBP), physical inactivity, office ergonomics, hypertension, nutrition, respiratory, and multi-topic. The number of the included studies based on the year of publication, topic and country is given in Figs. 2 and 3, and Fig. 4 respectively.

### Sedentary behaviors

A sedentary lifestyle is a significant public health concern in modern society [32, 35, 46, 47], as it can lead to poor physical and mental health [48–50] and the development of serious diseases such as cancer, obesity, metabolic syndrome, type 2 diabetes, and cardiovascular disease [51–59]. Sedentary behavior is defined as any waking behavior (sitting, reclining, or lying posture) that consumes 1.5 metabolic equivalents of energy [52] and is prevalent in office settings [60–64]. Inactive activities account for nearly 65–82% of working time in industrialized countries [64–66], and 54–77% of office workers sit all day [66–68]. To address the negative consequences of sedentary behavior in the workplace, effective interventions must be designed, including encouraging desk-based employees to spend at least 2–4 h standing or doing light activity, taking regular breaks from sitting [69], considering environmental factors [70, 71]., addressing concerns about productivity [72], and increasing awareness among employees and employers through training programs [73]. E-training has been used in [36, 73–76] to reduce sedentary behavior and address the mentioned challenges.

A theory-driven, web-based, computer-tailored application called Start to Stand has been developed to reduce sedentary behavior at work [36]. The application includes both mandatory and optional components. In the mandatory component, personalized advice on how to interrupt and reduce sitting was provided, and the optional component has five non-committal sections: interruptions in sedentary behaviors, replacing sedentary behaviors with standing, sedentary behaviors during commuting, sedentary behaviors during work breaks (e.g., lunch), and developing a sedentary behavior change action plan that motivates participants to achieve their objectives by creating an action plan. The application uses various theories, such as the Theory of Planned Behavior [77], self-determination theory [78, 79], and self-regulation theory [80], to provide recommendations to users. In order to evaluate the implemented application, the accessibility of participants and the acceptability were reported. One hundred and twelve employees from public city service were invited to participate in the study. The feasibility test showed that education, employment status, level of breaks at work, and attitudes towards interrupting sitting at work were influential in requesting advice, and 39% of participants requested at least one non-committal section from the optional component. The acceptability test revealed that most participants found the advice interesting, relevant, and motivating. The majority of participants (98.0%) reported that they had reduced their sedentary behavior or intended to do so.

The effects of the *Start to Stand* application [36] have been evaluated among Flemish employees in a field-based approach [75]. In order to assess a Randomized Controlled Trial (RCT) study designed with three groups; tailored group: the participants received the Start to Stand application, generic group: participants received a web-based application that provided general information on reducing or interrupting workplace sitting, and control group: participants did not receive any intervention. Two hundred thirteen employees participated in the intervention (tailored group n = 78, generic group n = 84, and control group n = 51). Outcomes were measured at baseline, one month, and three months after the intervention (follow-ups). Results showed a statistically significant difference in total workday sitting, sitting at work, other leisure time sitting, and break at work in the intervention group compared to the other two groups over time.

The dissemination evaluation of the Start to Stand application [36] in the Finnish population was done by [73]. The Reach, Effectiveness, Adoption, Implementation, and Maintenance framework (RE-AIM framework) [81, 82] has been used for this proposal, which evaluates the potential public health impact of behavioral interventions from five perspectives ‘reach’, ‘efficacy/effectiveness,’ ‘adoption,’ ‘implementation,’ and ‘maintenance.’ The main dissemination methods used were partner websites, emails, newsletters, and social media. Over the evaluation period (12 October 2016 until 6 February 2018), 6,906 unique users visited the site. The evaluation results of the RE-AIM framework components are reported as follows. Firstly, Reach, which compares the characteristics of the study population with the target population (population in Flanders), showed that participants significantly differed from the target population in terms of age, gender, Body Mass Index (BMI), and Physical Activity (PA). Secondly, Efficacy/Effectiveness refers to the application’s positive and negative consequences in optimal and real-world conditions, respectively. The results of this section are presented in [75]. Thirdly, Maintenance refers to a continuous effort to make the software available to the target audience. In this case, the website was available to the public from 12 to 2016 to 6 February 2018. Fourthly, Adoption assesses delivery staff and setting variables. This item is outside the scope of this article [74]. Lastly, Implementation refers to intervention fidelity and resources (cost and time) to be used to implement the system in a real-world setting. The time spent on the dissemination activities was about 25.6 h, and the cost paid to the staff to perform these activities was 845 EUR.

The effect of the action plan section of the Start to Stand application [36] on changing sedentary behavior was evaluated in [76]. Creating an action plan was a non-committal section of the optional component of the application. The study had two goals: to examine the characteristics of users who made action plans (Goal 1) and the content of those action plans (Goal 2). The action plans consisted of four parts: what behavior to change when the action plan is triggered, where the action plan takes place, how long/frequent the breaks from sitting, and the purpose of the action plan. Participants in this study (n = 1,701) were divided into two groups, participants who had made at least one action plan (Group 1, n = 231) and those who had not made any action plans (Group 2). The study found significant differences between the two groups in terms of age, sedentary behavior at work, and awareness of health risks related to prolonged sitting. The participants in Group 1 were older, more sedentary at work, and more aware of the negative consequences of prolonged sitting (Goal 1). Most generated action plans focused on breaks from sitting every 30 min and replacing sitting with periods of standing (Goal 2).

A mobile-based application has been developed by [73]; the study aims to investigate the feasibility of implementing the application to reduce sedentary behavior in desk-based office workers. The application provides feedback on prolonged sitting, educational facts, setting goals to reduce sitting and reminders to achieve these goals, and self-monitoring features. The intervention was designed for eight weeks, and a feasibility cluster randomized controlled study with three groups has been designed to evaluate the application: Mobile Application Group (MA Group): the participants received the application, Mobile Application and Sit-Stand Work Desk Group (MA + SSWD Group): in addition to the application, a sit-stand work desk (SSWD) also provided for the participants and control group. Fifty-six workers participated in the study that 20, 20, and 16 participants were allocated in the MA, MA + SSWD, and control group, respectively. The feasibility of the application was assessed using several measures, including recruitment and retention, engagement, intervention delivery, and acceptability. The recruitment of companies to participate in the study was challenging; however, the retention rate was high among the recruited groups. Regarding engagement, the MA group showed better response time than the MA + SSWD group, and they acknowledged the reminders more. However, technical issues and diminishing user engagement have compromised the delivery of the intervention. In terms of acceptability, most participants in both MA and MA + SSWD groups found the intervention appropriate. Nonetheless, the MA group reported higher satisfaction levels than the MA + SSWD group.

### Obesity

Obesity is a major risk factor for non-communicable diseases such as diabetes, cardiovascular diseases, and some cancers [83, 84]. Over 1.9 billion adults were overweight, and over 600 million were obese in 2014 [85]. Obesity is a significant global health challenge [86–89] and requires considerable healthcare resources to manage and prevent associated complications [90–93]. However, it is a modifiable disease [94, 95], and multicomponent behavioral interventions have been shown to significantly affect weight loss and obesity-related complications in adults [96–99]. E-training has been used as a behavioral intervention for weight loss in the workforce [37, 100–102]. An integrated and personalized mobile application for weight loss in work environments has been developed and evaluated by [37]. It includes a personalized diet prescription algorithm, a PA prescription algorithm, a convenient method of tracking daily diet and PA, and behavior change strategies for encouraging weight reduction to maximize user adherence. Weight reduction was the primary outcome. Thirty obese participants used the application for 12 weeks. They experienced a statistically significant mean weight reduction of 5.8%, along with improvements in secondary outputs such as anthropometric measures, metabolic profiles, fat measures, and bioimpedance measurements.

An online weight loss program for weight loss in work environments has been developed and evaluated by [100]. The percent weight loss and the achievement of clinical cut-points by class attendance were the primary and secondary outcomes, respectively. The intervention included a 10-week intervention focusing on the modification of eating habits (mindful eating, healthy eating, and stop-eating cues), medical considerations, weight loss, PA, weight maintenance, and self-monitoring. Data from 140,445 employees of different companies in the U.S. who used the program were analyzed, and class attendance and education level were found to be significantly correlated with percent weight loss. Evaluation of secondary outcomes determined that 71% of participants lost less than 3% of their starting weight, 16% achieved clinically beneficial weight loss (3-4% weight loss), and 13% achieved clinically significant weight loss (more than or equal to 5% weight loss).

The effectiveness of a weight loss mobile app based on WeChat is evaluated by [101] in a 6-month cohort study with 15,310 employees who worked in government agencies and enterprises in the Shunyi District of Beijing. The application was sent to the participants through messages on WeChat and allowed Participants to interact with others and receive expert feedback. Additionally, a weight loss unit rankings component was developed to motivate and encourage participants. Three thousand four hundred sixty-seven participants were in the control group, and 11,843 participants were in the intervention group. Those in the intervention group lost significantly more weight than those in the control group, with weight loss varying based on age (< 40 and ≥ 40) and educational level. The more active the intervention group members were, the more weight they lost.

An online lifestyle-intensive behavioral therapy-based intervention for weight loss and weight management for workers has been evaluated by [102]. The intervention was designed for 52 weeks, which included weekly 30-minute sessions that focused on education about PA, healthy eating, savvy food shopping, managing stress, sleep, and motivation. The content was based on the Centers for Disease Control and Prevention (CDC) DPP curriculum [103]. An entertaining television-like series was also shown during the classes to introduce a healthy lifestyle. In addition, personalized PA, meal plans, and exercise aids were provided for the first 16 weeks. In addition to the training section, there is a dashboard based on self-reported information and information on exercise. Sixty-nine thousand five hundred ninety-eight workers from 96 companies in the United States participated in this intent-to-treat cohort study. Participants were divided into three categories based on their attendance: those who attended at least one session, those who attended at least four active sessions (active participants), and those who completed the entire program (program completers). The participants who attended at least one session lost an average of 2.8% body weight, with 23% achieving 5% weight loss. The active participants lost an average of 3.5% body weight, with 29% achieving 5% weight loss. The program completers lost an average of 4.3% body weight, with 36% achieving 5% weight loss. Based on the results, gender, number of classes attended, obesity, and age are predictors of 5% and higher weight loss.

### Neck/shoulder pain/stiffness and low back pain

Musculoskeletal pain is one of the common medical complaints in the working population [104, 105]. Neck pain, shoulder pain, neck stiffness, shoulder stiffness, and LBP are the most important musculoskeletal pains in the workplace [25, 34]. Factors that contribute to the increase in work-related musculoskeletal pains can be divided into three categories: sedentary lifestyle in work factors [106–108], physical factors such as poor posture or muscle weakness [106], and psychological factors [109].

Based on moderate-to-strong evidence, exercise therapy can significantly relieve pain and improve musculoskeletal disease function [110–119]; however, adherence to prescribed exercises is a significant challenge [120, 121]. The lower the adherence to the exercise, the lower the effect of the treatment [122]. Therefore, adherence to the exercise should be considered in the design of interventions [123]. The attractiveness of the programs [122], expert feedback and interaction [124, 40, 122, 125–129], performance evaluation [122], support by experts [122, 123], and review sessions [123] are strategies that affect increasing adherence. In all three papers in this Sects.  [25, 34, 130], interventions have been designed to improve commitment to the prescribed exercises.

An exercise-based artificial intelligence (AI)–assisted Chatbot on the LINE application has been implemented and evaluated by [25]. LINE is the most popular social media application in Japan [131]. The Chatbot included interactive features to improve participants’ adherence to the exercise program. The degree of pain and the improvement of pain were the primary outcomes. A RCT was designed to evaluate the application, and 121 participants with either neck/shoulder stiffness/pain or LBP or both were randomly assigned to intervention (n = 61) and control (n = 62) groups. The intervention group received exercise and educational instructions for 12 weeks. The result showed that the intervention group had statistically significant improvements in pain levels and exercise adherence compared to the control group. The adherence rate in the intervention group was 92% during the intervention.

A smartphone-based exercise program has been implemented and evaluated by [34]. This program divided the office workers into four categories based on the type of neck pain and offered corresponding exercise programs for each category. For this purpose, an algorithm has been implemented to help the user determine the kind of neck pain using simple tests. A single-group repeated-measures study was designed to evaluate the application. The developed program was given to 23 office workers suffering from neck pain to follow an exercise program for 10–12 min per day, three days per week, for eight weeks. The primary outcomes evaluated in the study were pain intensity and functional disability, both of which showed significant improvements after the intervention. Some secondary outcomes were also evaluated in the study. Quality of life also improved significantly, while fear avoidance and cervical spine ranges of motion (cervical ROM) did not show significant changes. The patient satisfaction was 3.91 (5.0). The adherence rate was 91.85%. The average time duration per exercise session was 16.86 ± 7.38 min.

A tailored web-based exercise intervention for physically untrained office workers with sub-acute non-specific LBP has been evaluated by [130]. This study aimed to assess: the impact of an intervention on exercise-related behavior to improve LBP and its correlation with functional disability improvement. For the first purpose, participants’ health status was categorized into five categories (pre-contemplation, contemplation, preparation, decision/ action, and maintenance), and their behavior change was evaluated and updated after the intervention. Participants were then classified into three categories based on their behavioral changes: no change, negative behavioral change, or positive behavioral change. One hundred participants in the RCT study were equally divided into two intervention groups (intervention and standard care) and control groups (usual care only). The intervention group received educational content and exercises via the Internet, and after nine months, most participants were in maintenance status and willing to continue the program (first objective). For the second purpose, the intervention group’s positive behavioral change was statistically significant, and there was a strong correlation between the stage of change and functional disability levels.

### Physical inactivity

Physical inactivity is another occupational problem related to exercise that increases the risk of cardiovascular disease [132], cancer [133], musculoskeletal disorders [134], mental disorders and health conditions [135], and mortality [136]. It also leads to decreased quality of life [137] and daily activity performance [138, 139]. Therefore, the low level of PA in the workplace is a crucial health challenge [140], causing about 3.2 million deaths per year globally [136]. Based on the data collected from 122 countries, almost one-third of the adult population of these countries had a low level of PA [141]. Adherence to prescribed exercises is a challenge for promoting PA interventions in the workplace. Only 40–45% of studies reported positive effects [142, 143]. E-training has improved PA in the work environment [144, 145].

The impact of a pedometer-based intervention for the promotion of PA has been evaluated by [130]. This 6-week intervention is designed based on the socio-cognitive learning theory [146], the theory of planned behavior [147], and the Health Action Process Approach (HAPA) model [148]. The intervention incorporated gamification features such as quizzes that aim to improve participants’ knowledge on PA and general health, the ability for each participant to select up to three health goals per week from a pool of 60 predetermined options, and the participants can determine the number of steps as step goals. Participants can join the weekly team or even individually challenge and compare themselves with others based on the results of game elements. One hundred seventy-six workers participated in the RCT study, with 99 in the intervention group and 77 in the control group. Direct health promotion outcomes, such as PA-related knowledge, intentions, and self-efficacy, and intermediate health outcomes including, Time spent on vigorous PA per week, Time spent on moderate PA per week, and Time spent walking per week, were measured. The results showed significant differences in all direct health promotion outcomes and only in Time spent walking per week among intermediate health outcomes between the control and intervention groups.

The feasibility and effectiveness of a web-based program to increase PA have been evaluated by [145]. The intervention used a PA monitor (PAM) and a website to provide personalized PA recommendations. Participants could upload their scores on the PAM to the website and plan and evaluate their recommendations based on earned scores, preferences, and PA goals. A RCT study was designed with the participation of 102 workers from eight work sites in Amsterdam to evaluate the intervention. Participants were randomly assigned into control groups (n = 51) and intervention groups (n = 51). The intervention group received a PAM and Web-based tailored PA advice for three months. The control group received a brochure on increasing PA and brief general PA recommendations. Primary outcomes included time spent on light-intensity, moderate-intensity, and vigorous-intensity activity. Several secondary outcomes were also evaluated through measurements (body weight, body height, waist and hip circumference, BMI, etc.) or questionnaires (attitude, social influences, self-efficacy, etc.). The outcomes were evaluated before the intervention (Baseline), immediately after the intervention (Post-Test), and five months after the intervention (Follow-up). Results showed no significant differences in primary and secondary outcomes between the intervention and control groups at post-test and follow-up. Some process measures were also measured in the study. Most intervention group participants reported wearing the PAM regularly and setting personal goals on the website, but some found the advice unappealing, not personal enough, or impractical.

### Office ergonomics

Long-term computer use is crucial in office work [149] due to the increasing reliance on data-based technologies, which has made many office tasks heavily reliant on computers [150]. In the European Union, 50% of men and 45% of women use computers at work every day [151]. However, this prolonged computer use can lead to [152–156], eye strain [157–161], and psychosocial stress [162–164]. To mitigate these adverse effects, interventions at work fall into four categories: ergonomic workplace design, employee selection and placement, aggressive medical management, and employee training and education [165]. When the first three options are not always available, providing educational materials is a viable option [166–168]. E-training is used to reduce office ergonomics issues [38, 169].

Online office ergonomics training has been implemented and evaluated by [169]. The educational content includes six animated and interactive modules: Introducing office ergonomics, ergonomics awareness, ergonomics assessment/self-assessment, healthy work behaviors and Environment, how to Set up a Workstation, and Exercise at Work. Three hundred programmers or typists participated in this study, 250 of which were in the study group, and 50 were in the pilot group. The participants were assessed before training and immediately after training using knowledge and behavior assessments. A workplace interaction questionnaire and Rapid Upper Limb Assessment (RULA) were used to assess the behavior. Based on the score obtained from the questionnaire, the risk level of the participants was divided into four categories: Low, Mild, Moderate, High, and Very High. The RULA assesses working posture and identifies risks associated with the shoulder, hand, and wrist postures. Results showed statistically significant improvements in knowledge assessment scores, workplace interaction scores, RULA scores, and a decrease in complaints of health-related pains (Pain, Aching, Burning, Numbness, Tingling, Tenderness, Stiffness, and Cramp).

The position of the computer monitor relative to the eyes is an important factor in eye strain [158, 169], especially for those who wear glasses, particularly for presbyopic users [170]. Adjusting chairs can also reduce visual symptoms [171–173]. Therefore, the ergonomic design of a computer workplace has been introduced as an effective intervention [174].

A website has been evaluated by [38] to help employees adjust their computers in the work environment without expert support. The content presented on the website is divided into two parts: sitting position, table, keyboard and lighting, and the adjustment of the monitor in dependence on the kind of eyewear. Three categories of data were collected to evaluate the intervention: the table height, depth, width, and the height and width of the monitor and its inclination relative to vertical, the posture of participants during their natural working, gaze inclination, viewing distance, head inclination, and complaints (eye strain, headache symptoms, and musculoskeletal strain). Data were collected before the study and one week and five weeks after the intervention from 24 workers using computers. Participants were divided into two groups: those who were not presbyopic and those who were. The intervention lasted six weeks, and 23 of the 24 participants modified their computer workstations (6, 6, and 11 participants adjusted the monitor positioning, the chair, and both the monitor and the chair, respectively). The relationship between changes in ergonomics settings and complaints was investigated based on the reported complaints. The chair adjustment had a statistically significant effect on reducing musculoskeletal complaints. Lowering one’s gaze resulted in fewer visual complaints, which was statistically significant.

### Hypertension

Hypertension is a major risk factor for various cardiovascular diseases [175], and only a small percentage of people follow blood pressure treatment [176]. Worse cardiovascular outcomes and increase in treatment costs are negative consequences of poorly controlled hypertension [177, 178]. Interventions like weight loss [179, 180], exercise [181, 182], workplace health programs [183–185], and community education [186] are the most important category of interventions to reduce hypertension. Only one intervention [187] evaluated e-training in controlling hypertension in workers.

The effect of using a digital health intervention to control blood pressure has been investigated by [187] on 3,330 workers throughout the U.S. for more than a year. Participants were given educational and motivational materials and individualized care plans to help them manage their hypertension. Participants were divided into two categories: users: participants who visited the application five times or more during a year, and nonusers: participants who visited the application less than five times. Systolic Blood Pressure (SBP), Diastolic Blood Pressure (DSP), weight, and BMI were the primary outcomes in this study. Based on the reported result, the decrease in SBP, DSP, and BMI in users was significant compared to nonusers. Increasing login frequency was significantly associated with reducing SBP, DSP, weight, and BMI.

### Nutrition

A nutrient-poor diet is a major health risk for the workforce [188], leading to obesity, diabetes, and heart disease [189], harming work productivity [190–193], and absenteeism [191–193]. Therefore, investing in nutrition improves the health of society and national economies [190, 194]. Only one intervention [195] evaluated e-training in improving diets in the workplace.

Online self-paced training to improve knowledge and increase the healthy behaviors of construction workers has been developed and evaluated by [195]. The topics covered in this intervention are the importance of nutrition in occupational performance and health, obstacles to healthy eating, and solutions to promote healthy eating. It takes about 50 min to complete the training content. A pre-test and post-test design has been used to evaluate the intervention. Changes in knowledge and behavior were the primary outcomes. The changes were evaluated before the intervention (Baseline), immediately after the intervention (Post-training), and 12 weeks after the intervention (Follow-up). This intervention involved 62 apprentices from ten highway construction trades that were divided into two control and intervention groups. The intervention group provided online nutrition training, and the control group provided online content about positive thinking. The participants in the intervention group showed improvements in knowledge and positive behavior (reducing the consuming sugary snacks, reducing sugary beverages such as soda and coffee, reducing the eating of fast food, and increasing the consumption of fruits and vegetables) changes compared to the control group from baseline to post-training. However, these improvements were not maintained at the follow-up evaluation. In addition to the primary outcomes, accessibility was also evaluated. According to the published results, 70% of the participants in the intervention group reported they would highly recommend the training for others, and 75% believed that more training for occupational health and safety issues should be provided to the workforce.

### Respiratory

Occupational respiratory illnesses are one of the most important diseases of workers. Working conditions cause 15% of chronic obstructive lung diseases [196] and 15-23% of new-onset asthma in adults [197]. Education plays an important role in reducing these negative consequences. Only one intervention [198] evaluated e-training in respiratory safety.

A computer-based multimedia respiratory safety training has been evaluated by [198]. The content was prepared for two groups, young and old workers, according to working memory constraints and cognitive load theory [209–212] and the levels of learning. The educational content was designed in three formats: text (TXT), text with pictures/animations (TAP), and text with pictures/animations and audio narration (NAP). The educational content of all three types was the same and included: the employer’s responsibilities, how to clean respirators, how respirators work, etc. This intervention included 81 factory workers randomly assigned to one of three groups. The participants’ knowledge and ability to solve tasks relevant to the delivered information were examined immediately following the intervention. According to the findings, knowledge acquired by young and old participants is the same, regardless of educational content format. Still, the NAP group had the most significant effect on applying knowledge to the real world for both age groups. Additionally, the NAP group had a significant difference in problem-solving ability between young and old groups.

### Multi-topic

Training in previous studies was limited to one topic. Some studies cover various aspects of occupational safety and health and provide educational content on various topics. E-training has been used to learn several topics in [18, 199–202].

The effect of web-based health promotion programs on improving the productivity of employees has been assessed by [199], including weight management, chronic pain management, overcoming depression, and overcoming insomnia. The review did not include depression and sleep programs. There was no restriction on how many programs could be run concurrently. However, the reported results included employees who only participated in one program. Weight loss was the primary outcome of the weight management program, and pain intensity and pain unpleasantness were the primary outcomes of the chronic pain management program. Work productivity and activity impairment were assessed in addition to the primary outcome. The program included over 200,000 employees (weight: 218,081, pain: 7,145) and assessed primary outcomes at 30, 60, and 180 days following the interventions. Results showed that more than half of the participants experienced improved health status (reduction of pain and weight) compared to baseline, and work productivity and activity impairment scores were significantly lower than baseline at all time points. Furthermore, the findings revealed that improved worker health had a significant relationship with reduced productivity impairment.

Education and awareness, early detection, and disease management intervention have been evaluated by [200]. This workplace intervention aims to reduce the risk factors associated with metabolic syndrome. This multifaceted workplace intervention focuses on metabolic syndrome risk factors. The intervention is designed in the form of a website that comprises several sections. Firstly, the health risk assessment tool @live [203] is available to help users identify their risk factors. Secondly, on-site screening and clinical visits with registered nurses are offered. Thirdly, call-back interviews are conducted to address disease management, one of the most critical topics discussed during the phone calls. Finally, on-site educational programs are organized to enhance participants’ knowledge and awareness about the risk factors associated with metabolic diseases. The four risk factors for MetS (blood pressure, blood glucose, total cholesterol, and waist circumference) were primary clinical outcomes. Disease management was the secondary clinical outcome. The study involved 2,000 employees from over 30 worksites in British Columbia, and data was collected and analyzed over one year. The results showed a significant reduction in blood pressure and cholesterol levels, as well as a 15% reduction in the number of risk factors between baseline and six months after the intervention. The most important changes in disease management were medication changes, lifestyle changes, and visits to a family doctor.

A mobile-based educational intervention for U.S. dairy farm workers has been evaluated by [39], which aimed to address difficulties in providing occupational safety and health training due to an increase in the number of workers on the job, as well as immigrant workers with limited English proficiency. Providing a multilingual personalized learning environment based on the characteristics above is the solution presented in this study. The educational content consisted of general training on safety and health issues and specific training for three job categories in dairy farms: general and outside jobs, milker and calf caretaker, and feeder. After completing the first part, the participants followed the second part according to their job category. Kirkpatrick’s Four-Level Training Evaluation Model is used to evaluate training effectiveness [199], and 1,432 dairy workers from 40 farms participated in this study. The study found that participants reported good learning experiences (Level 1), and there was a statistically significant increase in their occupational health and safety knowledge before and after the training (Level 2). To evaluate Level 3, 3 months after the training, 9 out of 40 farms were randomly evaluated. 95% and participants applied the safety techniques learned in the workplace and stated they do their job more safely.

An online safety and health training intervention to increase knowledge and promote safe behavior of the young workforce has been evaluated by [198]. Promoting U through Safety and Health (PUSH) training is used in this study. PUSH training expands the content of Youth@Work [204, 205], a classroom-based curriculum to address the safety needs of young workers. PUSH develops this curriculum, including protection from workplace hazards, promoting health, and improving communication in the workplace. In addition to expanding the content, the format has changed from classroom-based to online format [206, 207]. One of the most important advantages of this course is covering a wide range of industries [208–211]. To evaluate this study, the knowledge of participants is measured before the intervention (Baseline), after the intervention (Post-training), and three months after the intervention (Follow-up). One hundred and twenty-four young workers participated in the pretest-posttest study. Based on the results, the increase in knowledge score in the post-training had a statistically significant difference from the baseline. The decrease in knowledge score after three months was significant compared to the post-training. In addition, the website’s likeability and acceptability, applying workplace training and changing behaviors was assessed. Participants reported that the training was interactive and informative, which improved their ability to identify and control hazards in the workplace, communicate with managers, and behave more safely.

The Massive Open Online Course (MOOC) Occupational Health that launched in 2015 and developed for Low- and Middle-Income Countries (LMIC) has been introduced by [201] to share knowledge on occupational safety and health. This study aims to explain the different characteristics of LMIC participants in MOOCs. Because there were no restrictions on using the MOOC, people from any country could register in the MOOC. The educational content included six modules, one module per week. The educational content includes: Module (1) basic concepts, Module (2) chemical and biological safety, Module (3) physical safety, Module (4) work-related diseases, Module (5) psychosocial safety, and Module (6) Care of the Worker. Each module contains 24–28 sections and takes approximately 4 h to complete. In addition, a discussion and feedback environment has been created for the participants. Of the 5,866 people who registered, 72.4% were from an LMIC, only 5% of the participants completed the training courses, and 23,547 comments were posted. 46.7% of the participants were females. Age is divided into 7 categories: <18, 18–25, 26–35, 36–45, 46–55, 56–65, and > 65 years. 0.3%, 12.1%, 38.3%, 26.7%, 11.1%, 7.8%, and 3% of participants were in the stated age groups, respectively. In terms of the employment sector, most participants (50.9%) were employed in the health and social sector. Regarding employment status, 57.8% reported that they are employed full-time, and 91.2% of the participants reported having education above secondary level. Improving career prospects, learning new things, and adding a fresh perspective to current work were the most important motivations for participating in this training course. The satisfaction of different parts of the MOOC (design and content) was 83–95%.

A digital toolkit to facilitate employees’ understanding of health screening at the workplace has been developed and evaluated by [18]. The development of this toolkit involved four stages: an online survey to examine employees’ views on health checks in the workplace and identify educational content and tool usage guide; stakeholder consultation to gather feedback and improve the guidance materials created in the first step; toolkit development and expert peer review to evaluate relevance, utility, and accessibility of the toolkit; and toolkit fidelity testing to evaluate the user experience, content relevance, utility, and accessibility of the implemented toolkit. The most important content suggested by stakeholders and experts is the business case for workplace health initiatives, the employer’s responsibility to provide a safe workplace, and providing information about occupational diseases and health. The implemented toolkit was given to 20 pilot employers, and fidelity (delivery and engagement) and implementation qualities (practicability, resource challenges, attitudes toward the toolkit, acceptability, usability, and cost) were assessed. The extent to which the intervention was delivered in accordance with the protocol (fidelity of delivery) and the extent to which the user engages with the content (fidelity of engagement) are measured in the fidelity assessment. According to the reported results, the toolkit had high fidelity of delivery and engagement. Based on the results of the evaluation of implementation qualities, a score is calculated for each item in implementation qualities, and the toolkit achieved the predetermined success rate in all items except the cost.

## Discussion

Educational program implementation necessitates both financial and human resources. Due to limited resources and infrastructure in developing countries, allocating resources for training is not a priority for managers, making providing effective training difficult. These constraints are less severe in developed countries, but because of the competitive environment and rising costs, managers must prioritize resource optimization to reduce costs.

Because of its benefits, e-training has been introduced as an alternative method of traditional learning in a variety of fields. E-training has been considered as an alternative to classroom-based occupational health and safety training methods in the last decade [20, 36–39]. As illustrated in Fig. 2, e-training has received a lot of attention since 2013, and the number of published studies has almost always increased.

The most important features in reducing costs in e-training programs are the ability to create content once and reuse it multiple times, access to content at any time and from any location, and the presence of infrastructures to simulate classroom-based training activities such as discussion forums [18, 33]. In addition, personalization of educational content based on needs, preferences and concerns, which are expensive to provide in class-based training, can increase the richness of educational content [20, 36–39].

In addition to affecting workforce health, effective classroom-based training fosters a sense of usefulness and importance in the workforce. As a result, it can boost work productivity, which is one of the most effective competitive advantages in industries. Concerning the distinction between classroom-based training and e-training, few studies examined the relationship between e-training and work productivity; more studies should be designed to investigate this relationship.

The difference in the number of studies conducted in developing and developed countries is significant. Out of 25 studies, 23 studies were conducted in developed countries (USA, Australia, Korea, UK, Germany, Belgium, Japan, Norway, Canada, Spain, and Netherlands) and only 2 studies were conducted in developing countries (Turkey and China). Due to the contextual differences, the results obtained in developed countries are not valid for developing countries. Therefore, there is a need for developing countries to conduct more research.

Occupational health and safety training covers a wide range of topics: chemical safety, electrical safety, fire safety, machine guarding safety, noise safety, lighting safety, etc. But educational interventions support a limited range of these topics. Although sedentary behaviors, obesity and physical inactivity, and office ergonomics problems are very important, interventions should also be designed to evaluate the impact of e-training on other topics.

Paying attention to the limited occupational safety and health topics has caused the effect of e-training to be investigated only in a narrow category of jobs. For example, sedentary behaviors and office ergonomics interventions focus on office workers. On the contrary, the audience of chemical safety or machine guarding safety training includes mostly workers who work in production lines and non-office environments. Considering the significant differences in the work environment, work activities, and capabilities of workers in different jobs, there is a need to design interventions to evaluate the impact of e-training in all topics. The lack of balance in the coverage of occupational health and safety topics prevents providing a big picture of the impact of e-training.

One of the most significant benefits of e-training is the ability to personalize content based on the needs, preferences, and concerns of the learners [20, 36–39]. One of the most difficult challenges for content personalization is the high diversity of learner characteristics in occupational safety and health training compared to other environments (such as university education). The most important differences are age, education level, work activities and work environments, language, ethnicity, race, familiarity with information technology, and prior knowledge. Often, workers, from newly hired young workers to older people nearing retirement, work alongside each other. Additionally, the workforce may have differing levels of education, ranging from elementary to higher education. Work activities and work environments vary greatly, from heavy manual work in high-risk environments to light office activities in low-risk environments. The responsibility for injury also differs among workers. In countries with a large immigrant workforce, language proficiency, ethnic differences, and racial differences can present significant challenges when providing educational programs. Familiarity with information technology is another area where there are differences among workers, as the degree of mastery and availability of tools such as phones, tablets, and internet access varies. Finally, workers’ prior knowledge may differ widely due to variations in completed occupational and health training programs. Due to the high diversity of learner characteristics, there is a need for occupational health and safety trainings to provide higher degrees of personalization.

The cost of implementation plays a significant role in managers’ decisions to implement interventions, particularly in developing countries. Educational interventions can increase costs in two ways: by producing educational content with complex and expensive technologies such as virtual reality [212–214] and augmented reality [215–217], and by using expensive intervention design tools such as ActivPAL [80], PAM [145], and SSWD [73]. ActivPAL recorded total sitting time awake, sitting at work, standing at work, and breaks at work. It is an inclinometer that can distinguish periods of sitting or lying from standing and assess breaks from sitting in adults [80]. The PAM is a device for recording PA (pedometer, accelerometer and etc.) automatically [145]. When these interventions are implemented in real-world settings, the costs rise exponentially. Because rising costs are a major impediment to implementing interventions in the real world, effective low-cost interventions should be developed. According to the reviewed interventions, simple content creation methods are also highly effective.

Some workers with special circumstances, such as the elderly [218–220], those with physical disabilities [221], immigrants [222–224], and others [225], have received less attention in studies. Because a number of workers are usually working in a shared working environment, the error of one worker may result in injury to other workers. As a result, there is a need to provide occupational health and safety training to all workers while taking into account their individual and social differences.

The use of social networks to implement educational interventions has received a lot of attention. Interventions based on WeChat [101], WhatsApp [33], and LINE [131] were designed for this review. Accessibility and the absence of the need to install additional software are two significant advantages of using social networks in educational interventions. Because these tools are intended for public discussions, they are not ideal for educational interventions. Of course, they can meet many of the needs of researchers.

Because confidential information about learners may be transferred during health education interventions, the use of social networks should be used with greater caution. Creating infrastructure, such as designing questionnaires, developing follow-up capabilities, and implementing structures to protect personal data, can greatly assist researchers in designing educational interventions. These features are also available on other websites, such as YouTube.

Two important aspects that receive little attention in studies are cost-effectiveness analysis and examining interventions in real-world settings. Real environments differ greatly from controlled environments. These distinctions can be seen in areas such as cost management. The difference in evaluation measures between the interventions performed in the controlled environment and the real environment can be seen in this review. Although lessons learned in controlled environments can increase the percentage of success in real-world situations, evaluating interventions in real-world situations provided a more complete picture of their effectiveness.

Occupational diseases and injuries typically involve multiple risk factors. For example, extensive research has been conducted to identify the risk factors of obesity. Some common risk factors for obesity include physical inactivity [226–229], unhealthy diet [230, 231], education level [232–236], length of sleep time [227, 228, 237], and stress/depression [230, 232, 234, 237]. As part of some research studies, efforts have been made toward categorizing risk factors. For instance, in [232], the authors have classified risk factors into four distinct categories: individual factors such as genetic factors (e.g., sex, ethnicity), depression, etc., social factors including family influences (e.g., marriage), peer influences, etc., lifestyle/behavioral such as food consumption (e.g., energy intake), PA, etc., environmental factors including community characteristics (e.g., rural-urban, access to unhealthy food options, crime), state policies, marketing, etc.

### Limitations

To effectively address health issues in educational interventions, it is necessary to take a comprehensive and holistic approach that considers all relevant factors. For example, studies surrounding obesity tend to prioritize low activity levels as a risk factor, while other areas may go unaddressed. We need to broaden our perspective and include a wider range of factors when designing and implementing occupational safety and health education interventions.

Because our search was confined to two databases and articles written in English, it is probable that some relevant articles were overlooked. This systematic review also omitted studies that employed e-training for Corona epidemic research. Second, we removed interventions related to psychological risk variables from our analysis. Taking these aspects into account can provide a complete picture of the success of occupational health and safety training interventions, in future studies. Finally, the studies in this systematic review used various assessment methodologies and outcomes, making comparison impossible.

## Conclusion

In this study, the literature review focuses on the possible impact of e-trainings on occupational safety and health. Technology has transformed how we approach training programs, and e-training has been proven to have various advantages in terms of flexibility, accessibility, and cost-effectiveness. The findings have demonstrated that e-trainings can be an effective tool for improving knowledge and skills among workers, which can ultimately lead to a reduction in workplace accidents and injuries. Additionally, e-training platforms can allow employers to track employee progress and ensure that training requirements are met. Further research is needed to explore this topic in greater depth and to identify best practices for implementing e-training programs in various industries.

## Data Availability

The datasets used and/or analyzed during the current study are available from the first author on reasonable request.

## Appendix

**Table 1:** Included studies in systematic review

First Author (Year) [Reference]	Purpose of study	Study design	Unit of allocation	Primary/secondary outcomes	Country	Platform
Wallen (2006) [199]	Respiratory safety	N/A	Workplace	Primary outcomes: Knowledge, Solve respiratory problem in workplace	USA	Website application
Slootmaker (2009) [143]	Feasibility and effectiveness of a website application to increase physical activity	Randomized controlled trial	Workplace	Primary outcomes: minutes per week spent on light-intensity, moderate-intensity, vigorous-intensity (physical activity levels) and time spent sedentary Secondary outcome: aerobic fitness (VO_2max_), body composition (weight, BMI, sum of skin folds, body fat, waist circumference) and determinants of playing sports (attitude, social influence, self-efficacy, intention) Process outcomes: Log-in frequency to the website, mean uploaded score to the website, appreciation of website, wore the accelerometer, set personal goal on website, entered favorite activities on website, read personalized advice on website, found advice on website appealing	Netherlands	Website application
Tarride (2011) [205]	An education and awareness, early detection and disease management (Heart disease, Stroke, Diabetes)	N/A	Workplace	Primary outcome: blood pressure, blood glucose, total cholesterol, and waist circumference Secondary outcome: disease management	Canada	Website application
Silberman (2011) [204]	Improving the productivity of employees (weight management program, chronic pain management program)	N/A	Individual	Primary outcome 1:weight loss Primary outcome 2: pain intensity and pain unpleasantness Secondary outcome: Work productivity and activity impairment	USA	Website application
del Pozo-Cruz (2013) [128]	Sub-acute non-specific low back pain	Randomized controlled trial	Individual	Improving the exercise-related behavior to improving LBP and the correlation this improvement with the improvement of functional disability	Spain	Website application
Meinert (2013) [168]	Eye problems (ergonomic design of a computer workplace)	Randomized controlled trial	Individual	Participants modified their computer workstations	Germany	Website application
Dalkılınç (2014) [167]	Office Ergonomics	N/A	Individual	Knowledge and risk level of the participants behavior number of complaints	Turkey	Website application
De Cocker (2015) [72]	Reduce sedentary behavior	Feasibility test Acceptability test	Workplace	Feasibility and acceptability	Belgium	Website application
Lee (2016) [36]	neck pain	Single-group repeated-measures	Individual	Primary outcome: pain intensity and functional disability Secondary outcome: quality of life, fear avoidance, and cervical spine ranges of motion Applicability outcomes: patient satisfaction and adherence	Korea	Mobile-based application
De Cocker (2016) [74]	Reduce sedentary behavior	Randomized controlled trial	Individual	Primary outcome: total workday sitting, total no-workday sitting, sitting at work, sitting on the bus, television viewing, personal computer use, other leisure time sitting, total sitting time awake, sitting at work, standing at work, and breaks at work.	Belgium	Website application
Dadaczynski (2017) [142]	The promotion of physical activity	Randomized controlled trial	Individual	Primary outcome: physical activity related knowledge, intentions, and self-efficacy Intermediate health outcomes: time spent vigorous physical activity per week, time spent moderate physical activity per week, and time spent walking per week	Germany	Website application
He (2017) [98]	Weight loss	N/A	Workplace	Primary outcome: weight loss	China	Mobile-based application based on WeChat
Senecal (2018) [168]	Control blood pressure (Hypertension)	N/A	Individual	Primary Outcome: SBP, DSP, BMI, weight	USA	Website application and Mobile-based application
Cocker (2018) [73]	Dissemination evaluation of reduce sedentary behavior application	Dissemination evaluation	Individual	Primary outcome: reach, efficacy/effectiveness, adoption, Implementation, and maintenance	Australia	Website application
Rodriguez (2018) [206]	Injuries in dairy farm	N/A	Workplace	Primary outcome: learning experiences, occupational health and safety knowledge, worker safety behavior	USA	Mobile-based application
Horstman (2018) [99]	Weight loss and weight management	Cohort study	Individual	Primary outcome: Weight loss	USA	Website application
Rohlman (2018) [196]	Improve knowledge and increase healthy behaviors (Nutrition)	Pre-test and post-test	Workplace	Primary outcome: knowledge, behavior (the consuming sugary snacks, consuming sugary beverages, eating fast food, eating meals from home, consuming of fruits and vegetables, servings of caffeine), accessibility	USA	Website application
Han (2019) [96]	Weight loss	N/A	Individual	Primary outcome: weight reduction Secondary outputs: anthropometric measures (weight, waist circumference, BMI), metabolic profiles(SBP, DBP, fasting plasma glucose, HbA_1c_, total cholesterol, triglyceride, HDL cholesterol, LDL cholesterol, Non-HDL cholesterol, AST, ALT), fat measures (CT fat ratio, visceral fat, Subcutaneous fat), and bioimpedance measurements (lean body mass, body fat)	Korea	Mobile-based application
Aryal (2019) [207]	General training	Pre-test and post-test	Individual	Primary outcome: Knowledge, likeability and acceptability, applying training in the workplace and changing behaviors	USA	Website application
De Cocker (2019) [75]	The effect of action plan on changing sedentary behavior	N/A	Individual	Examine the characteristics of users who created action plans and the content of action plans to reduce sedentary behavior.	Australia	Website application
Earnest (2019) [97]	Weight loss	N/A	Individual	Primary outcome: percent weight loss, clinical cut-points weight loss	USA	Website application
Blake (2020) [18]	General training	N/A	Individual	Primary outcome: fidelity (delivery and engagement) and implementation qualities (practicability, resource challenges, attitudes toward the toolkit, acceptability, usability, and cost)	UK	Website application
Tronstad (2020) [208]	General training			Primary outcome: explain the different characteristic of the LMIC participants	Norway	Website application
Anan (2021) [25]	Neck/shoulder pain/stiffness and low back pain	Randomized controlled trial	Individual	Primary outcome: subjective assessment of average level of pain and whether there was a pain improvement	Japan	Mobile-based Chabot on the LINE
Stephenson (2021) [71]	Reduce sedentary behavior	Feasibility cluster randomized controlled	Individual	Primary outcome: recruitment and retention, engagement, intervention delivery, acceptability	UK	Mobile-based application
